# Endotype-informed therapy and effects on illness perception and psychological health in patients with chest pain and no obstructive coronary artery disease

**DOI:** 10.1093/ehjqcco/qcag081

**Published:** 2026-06-04

**Authors:** Conor P Bradley, Gemma McKinley, Vanessa Orchard, Christina Tiller, Beth Stanley, Daniel Ang, Andrew J Morrow, Robert Sykes, Pamela Gildea, Maria Petty, Richard Brogan, David Carrick, Damien Collison, Hany Eteiba, Angie Ghattas, Richard Good, Francis Joshi, Mitchell Lindsay, Peter McCartney, James McGowan, Ross McGeoch, Keith Robertson, Paul Rocchiccioli, Aadil Shaukat, Stuart Watkins, Peter Kellman, Alex McConnachie, Colin Berry

**Affiliations:** British Heart Foundation Glasgow Cardiovascular Research Centre, University of Glasgow, 126 University Place, Glasgow G12 8TA, UK; Department of Cardiology, Golden Jubilee University National Hospital, Clydebank G81 4HX, UK; Robertson Centre for Biostatistics, University of Glasgow, Scotland G12 8TB, UK; Department of Cardiology, Golden Jubilee University National Hospital, Clydebank G81 4HX, UK; Internal Medicine III, Cardiology & Angiology, Innsbruck Medical University, 6020 Innsbruck, Austria; Robertson Centre for Biostatistics, University of Glasgow, Scotland G12 8TB, UK; British Heart Foundation Glasgow Cardiovascular Research Centre, University of Glasgow, 126 University Place, Glasgow G12 8TA, UK; Department of Cardiology, Golden Jubilee University National Hospital, Clydebank G81 4HX, UK; British Heart Foundation Glasgow Cardiovascular Research Centre, University of Glasgow, 126 University Place, Glasgow G12 8TA, UK; Department of Cardiology, Golden Jubilee University National Hospital, Clydebank G81 4HX, UK; British Heart Foundation Glasgow Cardiovascular Research Centre, University of Glasgow, 126 University Place, Glasgow G12 8TA, UK; Department of Cardiology, Golden Jubilee University National Hospital, Clydebank G81 4HX, UK; Department of Cardiology, Golden Jubilee University National Hospital, Clydebank G81 4HX, UK; Department of Cardiology, Golden Jubilee University National Hospital, Clydebank G81 4HX, UK; Department of Cardiology, Golden Jubilee University National Hospital, Clydebank G81 4HX, UK; Department of Cardiology, University Hospital Hairmyres, East Kilbride G75 8RG, UK; Department of Cardiology, Golden Jubilee University National Hospital, Clydebank G81 4HX, UK; Department of Cardiology, Golden Jubilee University National Hospital, Clydebank G81 4HX, UK; Department of Cardiology, Golden Jubilee University National Hospital, Clydebank G81 4HX, UK; British Heart Foundation Glasgow Cardiovascular Research Centre, University of Glasgow, 126 University Place, Glasgow G12 8TA, UK; Department of Cardiology, Golden Jubilee University National Hospital, Clydebank G81 4HX, UK; Department of Cardiology, Golden Jubilee University National Hospital, Clydebank G81 4HX, UK; Department of Cardiology, Golden Jubilee University National Hospital, Clydebank G81 4HX, UK; Department of Cardiology, Golden Jubilee University National Hospital, Clydebank G81 4HX, UK; Department of Cardiology, University Hospital Ayr, Ayr KA6 6DX, UK; Department of Cardiology, University Hospital Hairmyres, East Kilbride G75 8RG, UK; Department of Cardiology, Golden Jubilee University National Hospital, Clydebank G81 4HX, UK; Department of Cardiology, Golden Jubilee University National Hospital, Clydebank G81 4HX, UK; Department of Cardiology, Golden Jubilee University National Hospital, Clydebank G81 4HX, UK; British Heart Foundation Glasgow Cardiovascular Research Centre, University of Glasgow, 126 University Place, Glasgow G12 8TA, UK; Department of Cardiology, Golden Jubilee University National Hospital, Clydebank G81 4HX, UK; Medical Signal and Imaging Processing Program, National Heart, Lung and Blood Institute, Bethesda, MD 20892, USA; Robertson Centre for Biostatistics, University of Glasgow, Scotland G12 8TB, UK; British Heart Foundation Glasgow Cardiovascular Research Centre, University of Glasgow, 126 University Place, Glasgow G12 8TA, UK; Department of Cardiology, Golden Jubilee University National Hospital, Clydebank G81 4HX, UK

**Keywords:** Angina with no obstructive coronary artery disease (ANOCA), Coronary microvascular dysfunction, Cardiovascular magnetic resonance (CMR) imaging, Illness perception, Psychological health

## Abstract

**Aims:**

Patients with angina and no obstructive coronary artery disease (ANOCA) experience persistent symptoms, adverse illness perceptions, and psychological distress, often driven by diagnostic uncertainty. We evaluated whether non-invasive stress perfusion cardiovascular magnetic resonance (CMR), used to define ischaemic endotypes and guide targeted therapy, improves illness perception and psychological health compared with standard angiography-guided care.

**Methods and results:**

The Coronary microvascular angina Cardiac Magnetic Resonance imaging (CorCMR) trial was a prospective, multicentre, randomized controlled superiority trial. Patients with chest pain undergoing invasive coronary angiography showing no obstructive coronary artery disease were randomized 1:1 to CMR-guided endotype-specific management (intervention) or standard angiography-guided care (control). Illness perception was assessed using the Brief Illness Perception Questionnaire (BIPQ). Psychological health was assessed using the Patient Health Questionnaire-4 (PHQ-4). Assessments occurred at baseline, 6 months, and 12 months. A total of 250 participants were randomized (126 control, 124 intervention; mean age 63.3 years, 50.4% female). At 6 months, BIPQ scores were significantly lower in the intervention vs. control group [adjusted mean difference −8.67 (95% CI −12.04, −5.30); *P* < 0.001], with sustained benefit at 12 months [adjusted mean difference −10.80 (95% CI −14.27, −7.33); *P* < 0.001]. PHQ-4 scores were also significantly reduced at 6 months [adjusted mean difference −0.88 (95% CI −1.19, −0.57); *P* < 0.001] and 12 months [adjusted mean difference −0.97 (95% CI −1.29, −0.65); *P* < 0.001].

**Conclusion:**

In patients with ANOCA, non-invasive CMR-guided endotyping linked to targeted therapy resulted in substantial and sustained improvements in illness perception and psychological health, highlighting the importance of diagnostic clarification in addressing the psychological burden of this condition.

## Structured graphical abstract

**Structured graphical abstract:**
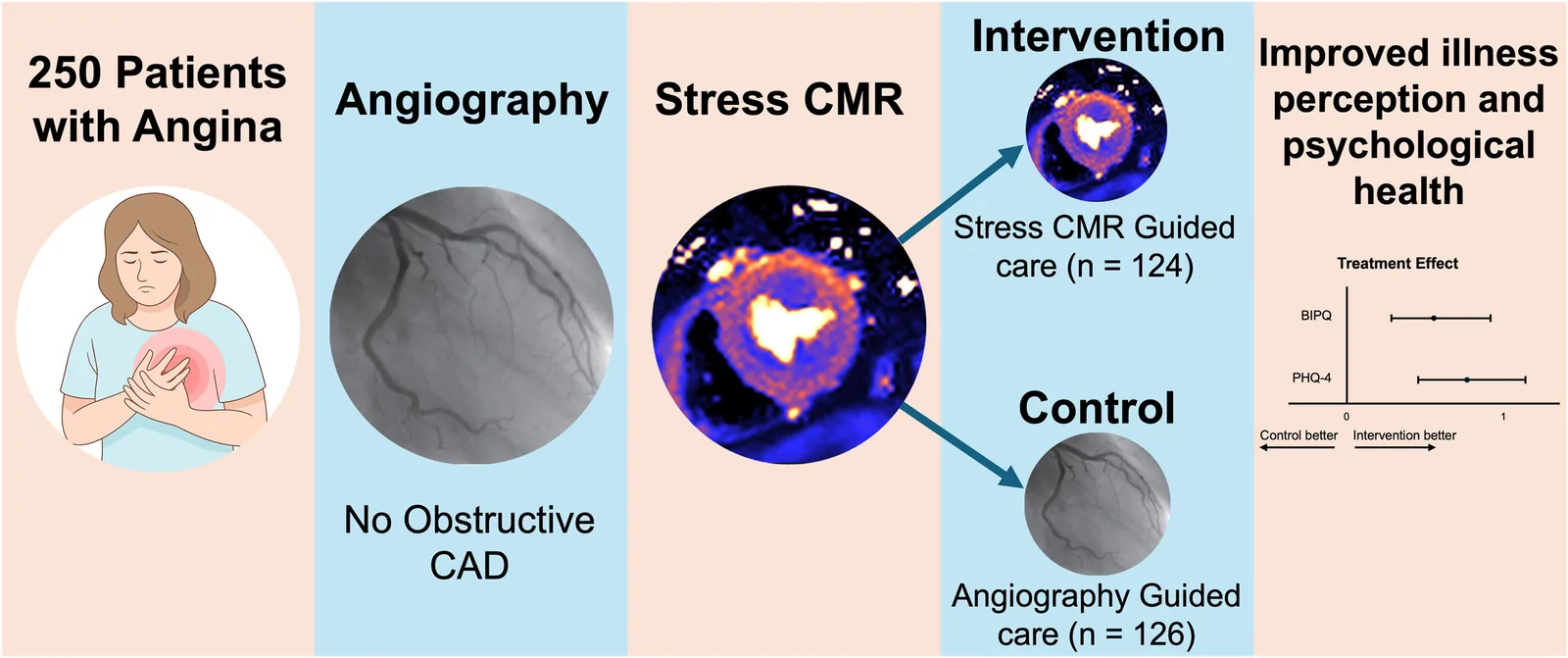
The CorCMR Trial Design and Health Related Outcomes

Key Learning Points What is already known

- Microvascular angina and vasospastic angina are prevalent causes of chest pain. Anxiety and depression are commonly associated features, and women are disproportionately affected.
- Coronary angiography clarifies the presence and severity of coronary atherosclerosis, but this test is insensitive for microvascular disease.
- Technical advances in cardiovascular magnetic resonance (CMR) imaging now enable mapping of myocardial blood flow (mL/min/minute)

What this study adds

- In this randomized controlled trial involving people with an empirical diagnosis of non-cardiac chest pain after coronary angiography, compared with standard care, adenosine-stress CMR-guided endotype-specific management was associated with improvements in adverse illness perception and psychological distress at 6 and 12 months.
- The results indicate that a diagnostic strategy based on myocardial blood flow imaging leads to durable benefits on psychological well-being in patients with angina and no obstructive coronary arteries (ANOCA).

Take-home Message

- In patients with possible ANOCA, angiography-guided management may commonly result in a missed diagnosis of microvascular angina, promulgating a burden of illness, anxiety, and depression.
- A clinical strategy of functional testing following coronary angiography to identify and treat the true cause of angina improves how patients understand their diagnosis and related psychological well-being.

## Introduction

Chest pain is a common and often distressing clinical condition, yet up to half of patients referred for invasive coronary angiography or coronary computed tomography angiography for the investigation of suspected chest pain of cardiac origin, that is, angina, have no obstructive coronary artery disease (ANOCA).^1^ In this population, symptoms are frequently attributed to non-cardiac causes despite ongoing chest pain, repeated healthcare encounters, and impaired quality of life.^2,3^ Coronary microvascular dysfunction and other vasomotor disorders are now recognized as prevalent and prognostically relevant causes of angina in the absence of obstructive coronary disease, but they often remain undiagnosed due to the reliance on anatomical assessment of epicardial coronary arteries alone.^4,5^

Beyond physical symptoms, patients with ANOCA may experience a substantial psychological burden. Persistent chest pain in the absence of a clear diagnosis is associated with heightened anxiety, depressive symptoms, adverse illness perceptions, and reduced functional capacity.^2,3^ Illness perception—encompassing patients’ beliefs about the cause, consequences, controllability, and timeline of their condition—is a key determinant of symptom burden, health behaviours, and mental health outcomes in chronic disease.^6^ Negative illness perceptions have been linked to greater symptom persistence, reduced treatment adherence, and worse quality of life in patients with stable angina and chronic coronary syndromes.^2,3^

Diagnostic uncertainty may underpin health anxiety. When coronary angiography excludes obstructive disease without providing an alternative mechanistic explanation for symptoms, patients may perceive their condition as unexplained, uncontrollable, or misunderstood. This uncertainty can perpetuate maladaptive illness beliefs, exacerbate anxiety and depressive symptoms, and undermine confidence in medical therapy.^2^ The Coronary Microvascular Angina (CorMicA) trial demonstrated that accurate diagnosis of coronary microvascular dysfunction using coronary function testing during invasive management of suspected ANOCA is associated with improvements in symptoms and health-related quality of life, including illness perception.^7^ These results have recently been validated in the ILIAS ANOCA trial.^8^

Whilst contemporary guidelines give a Class I recommendation to the use of invasive coronary function testing in patients with suspected ANOCA,^9^ invasive coronary function testing is not widely adopted in clinical practice. For example, in the current multicentre study, only 5.6% of participants had coronary function tests as part of invasive management. Stress perfusion cardiovascular magnetic resonance (CMR) imaging enables non-invasive, quantitative assessment of myocardial blood flow and identification of coronary microvascular dysfunction and other ischaemic endotypes,^10,11^ and currently has a Class IIb recommendation.^9^ When incorporated into a structured diagnostic pathway, stress CMR has been shown to reclassify diagnoses and guide endotype-specific therapy in patients with suspected ANOCA.^12^ However, the extent to which endotyping influences illness perception and psychological health is uncertain.

The Coronary microvascular angina Cardiac Magnetic Resonance imaging trial (CorCMR) was designed to evaluate whether non-invasive stress perfusion CMR, used to guide endotype-specific management, improves patient-reported outcomes in patients with ANOCA.^12,13^ While improvements in angina severity and health status have been reported previously, the present pre-specified analysis focuses on two interrelated psychological domains: illness perception, assessed using the Brief Illness Perception Questionnaire,^6^ and psychological health, assessed using the Patient Health Questionnaire-4.^14^

We hypothesized that CMR-guided endotyping would result in more favourable illness perceptions and reduced symptoms of anxiety and depression compared with standard angiography-guided care.

## Methods

### Study design

The study design and primary outcome results have previously been published.^12,13^ This was a prospective, multicentre, parallel group, 1:1 randomized, controlled superiority trial. The protocol was approved by the West of Scotland Research Ethics Committee (reference 20/WS/0159). This analysis represents a pre-specified objective of the study, as defined in the protocol and public registration (NCT04805814).

### Population

Elective referrals for invasive coronary angiography for the investigation of suspected angina were prospectively screened at three National Health Service (NHS) Scotland hospitals (Golden Jubilee University National Hospital, University Hospital Hairmyres, and University Hospital Ayr). Eligible participants who had undergone standard invasive coronary angiography, including fractional flow reserve measurement when indicated, within 3 months, which excluded obstructive coronary artery disease, were invited to participate. The Scottish Index of Multi-deprivation (https://www.gov.scot/collections/scottish-index-of-multiple-deprivation-2020/) was used to measure relative deprivation across seven domains—Income, Employment, Health, Education, Access to Services, Crime, and Housing.

### Eligibility criteria

The inclusion criteria were:

1. age ≥18 years;
2. symptoms of angina or angina-equivalent informed by the Rose Angina questionnaire;
3. coronary angiography within 3 months with a plan for medical management.

The exclusion criteria were:

1. obstructive coronary artery disease i.e. a stenosis >70% in a single segment or 50–70% in two adjacent segments in a coronary artery >2.5 mm, or fractional flow reserve ≤0.80;
2. Coronary revascularization by percutaneous coronary intervention or coronary artery bypass graft surgery following the index angiogram;
3. Prior coronary artery bypass surgery;
4. An alternative diagnosis that would explain the angina e.g. anaemia, aortic stenosis, hypertrophic cardiomyopathy;
5. Contra-indication to contrast-enhanced CMR e.g. estimated glomerular filtration rate < 30 mL/min/1.73 m^2^;
6. Contra-indication to intravenous adenosine, i.e. severe asthma; long QT syndrome; second- or third-degree atrioventricular block and sick sinus syndrome;
7. Lack of informed consent.

### Enrolment

The Study Information Sheet and Consent form were provided to potentially eligible patients after the standard care coronary angiogram. Patients were invited to participate after the standard care diagnosis had been assigned. Patients who provided written informed consent attended a reference centre (Golden Jubilee University National Hospital) for non-invasive endotyping by stress perfusion CMR imaging.

### Invasive and non-invasive assessment of diagnosis

The initial diagnosis and management plan (usual care) were documented by the interventional cardiologist after coronary angiography and before randomization and CMR imaging. The initial diagnosis was assigned by the interventional cardiologist following completion of a standardized clinician questionnaire (see Supplementary material online, *Figure S1*).

All randomized participants subsequently underwent assessment of myocardial blood flow using adenosine stress perfusion CMR imaging coupled with in-line pixel mapping. All CMR scans were reported by an imaging cardiologist who was blinded to randomized group.

In participants randomized to the intervention group, CMR-derived myocardial blood flow was used to inform the final diagnosis. In contrast, participants assigned to the control arm received standard angiography-guided management (usual care), and CMR myocardial blood flow findings were not used to inform the final diagnosis.

Given the potential for CMR imaging to reveal clinically or prognostically significant abnormalities—such as valvular heart disease, hypertrophic cardiomyopathy, or extracardiac pathology—any relevant incidental findings were disclosed for all participants, irrespective of randomized group allocation. These findings were managed according to standard clinical practice and did not result in unblinding.

### Randomization, groups, and blinding

Participants who provided written informed consent and attended for CMR imaging were eligible for randomization. Randomization was performed at the time of attendance for stress CMR. Individuals who initially consented but withdrew prior to attending for CMR were not randomized.

Randomization occurred before image acquisition to minimize bias. A secure web-based system was used to assign participants in a 1:1 ratio to the intervention group, in which the final diagnosis incorporated stress CMR findings, or to the control group, in which CMR findings did not inform the final diagnosis. Blinding was maintained for all participants. Randomization used a minimization algorithm balancing study site, sex, prior myocardial infarction, angiographic evidence of coronary artery disease, diabetes status, and left ventricular systolic function (<55% vs. ≥55%). To reduce predictability, a random component was included whereby two participants in each block of 10 were allocated purely at random, with one assigned to each group.

Participants, radiographers, and clinical staff involved in routine care were blinded to group allocation and to CMR-derived myocardial blood flow measurements. All participants received a standard clinical CMR radiology report that did not disclose randomized group assignment. Clinical outcome assessors were also blinded. The effectiveness of blinding was prospectively documented in the electronic case report form. Blinding effectiveness at 12 months post-randomization was prospectively assessed and confirmed in all (*n* = 250) participants (see Supplementary material online, *Table S1*).

### Stress perfusion cardiovascular magnetic resonance imaging and analysis

The stress perfusion CMR imaging and reporting protocol have been previously described and published.^12,13^

### Definition of endotypes

Following standard care angiography, all participants were assigned a diagnostic endotype by the attending interventional cardiologist (obstructive coronary artery disease, microvascular angina, vasospastic angina, and non-cardiac chest pain). The initial diagnosis was determined by the interventional cardiologist using the clinical history, results of prior non-invasive investigations, and angiographic findings. Whilst acetylcholine provocation testing was not part of the study protocol, the clinician could still make a presumptive diagnosis of vasospastic angina based on the clinical history.

Following CMR imaging, the initial post-angiography diagnosis was systematically re-assessed for all participants by the imaging cardiologist, incorporating CMR-derived myocardial blood flow data and any clinically relevant incidental findings. Myocardial blood flow measurements were used to classify the underlying disease endotype in accordance with pre-specified diagnostic criteria.^10,11^ These classifications were recorded in the study research database.

In participants allocated to the intervention group, CMR findings were used to revise the final clinical diagnosis and to allocate individuals to specific diagnostic endotypes, including microvascular angina, vasospastic angina, non-cardiac chest pain, or obstructive coronary artery disease. In contrast, for participants in the control group, myocardial blood flow information was not applied to modify the post-angiography diagnosis, which remained guided by standard angiographic assessment.

### Medical management

Clinicians responsible for participants’ ongoing care received the radiology report documenting the final diagnosis following CMR imaging. After completion of the CMR examination and confirmation of diagnosis, the unblinded research cardiologist coordinating the protocol selected a pre-specified management strategy tailored to the assigned endotype, irrespective of randomized allocation. This strategy comprised pharmacological therapy and lifestyle interventions to optimize cardiovascular risk factors in accordance with contemporary guidelines.^9,15^ In the control group, the diagnosis was guided by coronary angiography. Whilst in the intervention group, the diagnosis was guided by stress perfusion CMR findings.

Management recommendations were communicated to primary and secondary care clinicians involved in follow-up. Treating clinicians were encouraged to up-titrate medical therapy to address persistent symptoms during follow-up. Clinical management was led by blinded usual-care teams rather than the research investigators, and all treatment adjustments were made at the discretion of the treating clinicians. Standardized correspondence outlining individualized management recommendations was provided to the general practitioner and cardiologist to facilitate optimization of antianginal therapy. Participants in the control group received guideline-directed medical therapy. Patients with newly diagnosed ischaemic heart disease were prioritized for referral to cardiac rehabilitation.

Clinically significant incidental cardiac or extracardiac findings identified on CMR imaging were disclosed, and subsequent management followed established standards of care.

### Patient-reported outcome measures

Patient-reported outcome measures (PROMS) were assessed at baseline, 6 and 12 months, with the latter scheduled as an in-person visit to the clinical research facility. The PROMS were completed at baseline before randomization, and when completing the questionnaires, the participants were unaware of the CMR findings or the randomized group.

Illness perception was assessed using the Brief Illness Perception Questionnaire (BIPQ).^6^ This is a short, validated nine-item questionnaire designed to rapidly assess the cognitive and emotional perceptions of illness. Eight items are rated on a 0–10 response scale and assess different dimensions of illness perception: consequences (perceived impact on life), timeline (expected duration), personal control (belief in ability to control the illness), treatment control (belief in treatment effectiveness), identity (symptom experience), concern (degree of worry), coherence (understanding of the condition), and emotional response (emotional impact). A ninth open-ended question asks patients to identify the three most important causal factors in their illness. The eight quantitative items are summed to create a total score ranging from 0 to 80, with higher scores representing more threatening or negative perceptions of illness.

Psychological health was assessed using the Patient Health Questionnaire-4 (PHQ-4).^14^ This is a brief, validated 4-item screening tool that combines the 2-item Generalized Anxiety Disorder scale (GAD-2)^16^ and the 2-item Patient Health Questionnaire depression scale (PHQ-2)^17^ to assess symptoms of anxiety and depression in clinical populations. Each item asks about the frequency of symptoms over the past 2 weeks and is scored on a 4-point Likert scale (0 = ‘not at all’ to 3 = ‘nearly every day’). The total score is scored between 0 and 12, with higher scores representing greater psychological distress.

### Statistical methods

The study design and sample size calculations have previously been described.^9^

The change in PROMs was compared between randomized groups using linear regression. The intervention effect estimate (adjusted between-group mean difference) was reported with a 95% confidence interval and *P*-value. Residual distributions were examined visually, and standard transformations were applied where necessary to improve model fit. Comparisons of other secondary and exploratory outcome variables were done using Fisher tests (categorical outcomes) or the Mann–Whitney U test (continuous outcomes) where appropriate.

Statistical analyses were conducted at the data centre (Clinical Trials Unit, Robertson Centre for Biostatistics, University of Glasgow) according to a pre-specified Statistical Analysis Plan and the intention-to-treat principle. The analyses were conducted using R Studio and R version 4.5.1 (R Foundation for Statistical Computing, Vienna, Austria).

## Results

Between 9 February 2021 and 18 August 2023, 273 outpatients who had undergone clinically indicated invasive coronary angiography for investigation of chest pain and had a report of no obstructive coronary artery disease were screened and provided written informed consent within 3 months of the angiogram.

Two hundred and fifty of these patients attended for CMR imaging and were randomized before the scan. One randomized participant was unable to complete the scan due to claustrophobia. Twenty-three patients who had provided consent did not attend for CMR imaging due to logistical and other reasons.

### Baseline characteristics

The baseline characteristics of the participants are described in *Table 1*. Two hundred and fifty participants (91.6%) were randomized (*n* = 126 control group, *n* = 124 intervention group). The mean age of participants in the trial was 63.3 years, and there was an even distribution by sex (50.4% female).

**Table 1:** Baseline demographic and clinical characteristics for the randomized population

Characteristic	All *n* = 250	Control *n* = 126	Intervention *n* = 124
*Demographics*			
Age (years), mean (SD)	63.3 (9.2)	64.0 (8.9)	62.5 (9.4)
Sex			
Male, *n* (%)	124 (49.6%)	60 (47.6%)	64 (51.6%)
Female, *n* (%)	126 (50.4%)	66 (52.4%)	60 (48.4%)
Ethnicity			
White, *n* (%)	241 (96.4%)	121 (96.0%)	120 (96.8%)
Asian, *n* (%)	8 (3.2%)	5 (4.0%)	3 (2.4%)
Black, *n* (%)	1 (0.4%)	0 (0.0%)	1 (0.8%)
BMI (kg/m^2^), mean (SD)	30.3 (5.1)	30.2 (5.4)	30.5 (4.7)
BMI ≥ 30 kg/m^2^, *n* (%)	128 (51.2%)	60 (47.6%)	68 (54.8%)
Waist circumference (cm), mean (SD)	97.1 (13.4)	96.2 (14.0)	98.1 (12.7)
Smoking status			
Current smoker, *n* (%)	21 (8.4%)	12 (9.5%)	9 (7.3%)
Former smoker, *n* (%)	83 (33.2%)	44 (34.9%)	39 (31.5%)
Never smoked, *n* (%)	146 (58.4%)	70 (55.6%)	76 (61.3%)
Previous coronary angiogram, *n* (%)	56 (22.4%)	29 (23.0%)	27 (21.8%)
Previous percutaneous coronary intervention, *n* (%)	41 (16.4%)	20 (15.9%)	21 (16.9%)
Previous myocardial infarction, *n* (%)	30 (12.0%)	17 (13.5%)	13 (10.5%)
Previous stroke or transient ischaemic attack, *n* (%)	11 (4.4%)	4 (3.2%)	7 (5.6%)
Hospitalization for chest pain, *n* (%)	105 (42.0%)	49 (38.9%)	56 (45.2%)
History of valve disease, *n* (%)	6 (2.4%)	2 (1.6%)	4 (3.2%)
Hypertension, *n* (%)	117 (46.8%)	61 (48.4%)	56 (45.2%)
Diabetes mellitus, *n* (%)	43 (17.2%)	22 (17.5%)	21 (16.9%)
Atrial fibrillation, *n* (%)	19 (7.6%)	10 (7.9%)	9 (7.3%)
Chronic obstructive pulmonary disease, *n* (%)	35 (14.0%)	16 (12.7%)	19 (15.3%)
Systolic blood pressure (mmHg), mean (SD)	138.5 (17.7)	136.8 (16.2)	140.1 (19.0)
Diastolic blood pressure (mmHg), mean (SD)	77.5 (10.0)	76.2 (8.7)	78.7 (11.1)
Charlson comorbidity index, mean (SD)	2.4 (1.4)	2.5 (1.4)	2.3 (1.4)
QRISK3 predicted 10-year risk of MI or stroke, mean (SD)	17.2 (12.1)	18.0 (13.2)	16.3 (10.9)
Preventive therapy			
Aspirin, *n* (%)	213 (85.2%)	103 (81.7%)	110 (88.7%)
Statin, *n* (%)	223 (89.2%)	109 (86.5%)	114 (91.9%)
Ezetimibe, *n* (%)	5 (2.0%)	1 (0.8%)	4 (3.2%)
Angiotensin converting enzyme inhibitor, *n* (%)	75 (30.0%)	47 (37.3%)	28 (22.6%)
Angiotensin receptor blocker, *n* (%)	23 (9.2%)	8 (6.3%)	15 (12.1%)
Angina medication			
Beta blocker, *n* (%)	196 (78.4%)	95 (75.4%)	101 (81.5%)
Calcium channel blocker, *n* (%)	78 (31.2%)	47 (37.3%)	31 (25.0%)
Nitrates, *n* (%)	119 (47.6%)	59 (46.8%)	60 (48.4%)
Nicorandil, *n* (%)	32 (12.8%)	20 (15.9%)	12 (9.7%)
Glyceryl trinitrate spray, *n* (%)	201 (80.4%)	100 (79.4%)	101 (81.5%)
*Clinical characteristics*			
Cholesterol and lipid profile			
Total cholesterol (mmol/L), mean (SD)	4.0 (1.0)	4.1 (1.1)	4.0 (1.0)
HDL cholesterol (mmol/L), median [Q1, Q3]	1.3 [1.1, 1.6]	1.3 [1.1, 1.6]	1.3 [1.1, 1.5]
LDL cholesterol (mmol/L), median [Q1, Q3]	1.8 [1.4, 2.4]	1.9 [1.4, 2.4]	1.8 [1.4, 2.4]
Triglycerides (mmol/L), median [Q1, Q3]	1.4 [1.0, 1.8]	1.4 [1.1, 1.9]	1.3 [0.9, 1.7]
HbA1C, median [Q1, Q3]	38.0 [36.0, 42.0]	38.0 [35.0, 43.0]	39.0 [37.0, 42.0]
Canadian Cardiovascular Society angina class			
Class 0: asymptomatic angina, *n* (%)	0 (0.0%)	0 (0.0%)	0 (0.0%)
Class I: angina with strenuous exertion, *n* (%)	28 (11.2%)	14 (11.1%)	14 (11.3%)
Class II: angina with moderate exertion, *n* (%)	128 (51.2%)	63 (50.0%)	65 (52.4%)
Class III: angina with mild exertion, *n* (%)	89 (35.6%)	47 (37.3%)	42 (33.9%)
Class IV: angina at rest, *n* (%)	5 (2.0%)	2 (1.6%)	3 (2.4%)
Rose Angina Questionnaire			
Rose angina, *n* (%)	135 (54.0%)	66 (52.4%)	69 (55.6%)
Rose Angina Grade I, *n* (%)	74 (55.2%)	42 (64.6%)	32 (46.4%)
Rose Angina Grade II, *n* (%)	60 (44.8%)	23 (35.4%)	37 (53.6%)
*Seattle Angina Questionnaire*			
SAQ summary, mean (SD)	51.1 (20.7)	53.0 (20.9)	49.2 (20.4)
SAQ angina frequency, mean (SD)	55.8 (25.5)	58.3 (25.3)	53.4 (25.5)
SAQ physical limitation, mean (SD)	53.6 (25.0)	53.4 (24.3)	53.9 (25.8)
SAQ quality of life, mean (SD)	43.5 (23.7)	46.4 (25.5)	40.5 (21.6)
SAQ stability, mean (SD)	51.1 (26.0)	51.2 (26.2)	51.0 (25.8)
SAQ treatment satisfaction, mean (SD)	66.6 (23.1)	68.7 (22.5)	64.4 (23.6)
*Quality of life (EQ-5D-5L)*			
Index score, mean (SD)	0.6 (0.3)	0.6 (0.2)	0.6 (0.3)
Visual Analogue Scale, median [Q1, Q3]	60.0 [45.0, 75.0]	60.0 [49.2, 75.0]	60.0 [40.0, 75.0]
*Non-invasive diagnostic stress testing*			
Any stress testing performed, *n* (%)	173 (69.2%)	87 (69.0%)	86 (69.4%)
Treadmill stress testing performed, *n* (%)	151 (87.3%)	74 (85.1%)	77 (89.5%)
Abnormal treadmill stress testing results, *n* (%)	80 (53.0%)	36 (48.6%)	44 (57.1%)
Stress SPECT performed, *n* (%)	23 (13.3%)	14 (16.1%)	9 (10.5%)
Abnormal stress SPECT results, *n* (%)	11 (47.8%)	6 (42.9%)	5 (55.6%)
Stress echocardiogram performed, *n* (%)	4 (2.3%)	2 (2.3%)	2 (2.3%)
Abnormal stress echocardiogram results, *n* (%)	3 (75.0%)	2 (100.0%)	1 (50.0%)
*Coronary CTA*			
Coronary CTA performed, *n* (%)	39 (15.6%)	22 (17.5%)	17 (13.7%)
Coronary CTA normal, *n* (%)	5 (12.8%)	4 (18.2%)	1 (5.9%)
Intermediate coronary artery disease, *n* (%)	20 (51.3%)	12 (54.5%)	8 (47.1%)
Obstructive coronary artery disease, *n* (%)	14 (35.9%)	6 (27.3%)	8 (47.1%)

Values are mean (SD), median [Q1, Q3], or *n* (%). *QRisk3 score.

BMI, body mass index; coronary CTA, computed tomography angiography; CVD, cardiovascular disease; Hb, haemoglobin; HDL, high-density lipoprotein; LDL, low-density lipoprotein; NYHA, New York Heart Association; SAQ, Seattle Angina Questionnaire; SD, standard deviation; SPECT, Single Photon Emission Computed Tomography; TIA, transient ischaemic attack.

Nineteen (5.6%) participants had undergone coronary function testing during the angiogram. There was a high prevalence of cardiovascular risk factors and preventative medications in keeping with an elevated 10-year risk of cardiovascular disease event (mean 17.2%). In the randomized population, antianginal therapies were commonly prescribed: beta-blockers (*n* = 196; 78.4%), long-acting nitrates (*n* = 119; 47.6%), and calcium-channel blockers (*n* = 78; 31.2%). Following randomization, CMR-guided management was associated with higher use of preventive therapies (85.5% vs. 67.5%; *P* = 0.001), and more targeted antianginal prescribing, including calcium channel blockers (43.5% vs. 27.0%; *P* = 0.008) and long-acting nitrates (56.5% vs. 32.5%; *P* < 0.001).

### Endotypes

In the diagnostic study involving stress perfusion CMR imaging, diagnostic reclassification occurred in 131 (53.0%, 95% CI: 46.6–59.3%, *P* < 0.001) participants. Final endotype diagnoses were: microvascular angina 127 (51.0%), non-cardiac chest pain 117 (47.0%), vasospastic angina 1 (0.4%), and other diagnoses 4 (1.6%). Diagnoses by randomized group are described in Supplementary material online, *Table S2*.

### Illness perception

**Figure 1:**
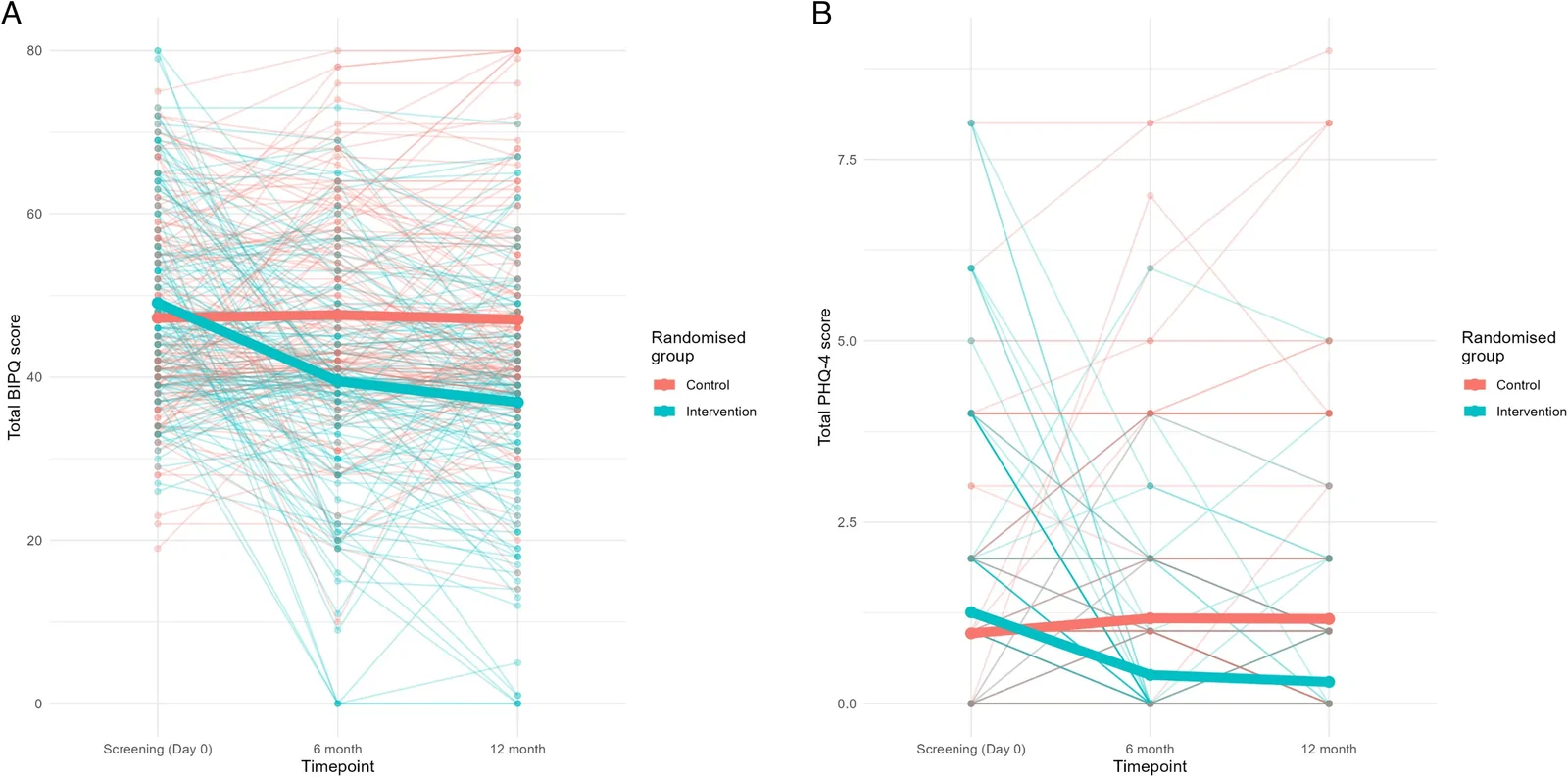
Illness perception and psychological health outcomes at baseline, 6 months, and 12 months. Spaghetti plots for (*A*) illness perception: Brief Illness Perception Questionnaire (BIPQ); higher scores represent more negative perceptions of illness, and (*B*) psychological health: Patient Health Questionnaire-4 (PHQ-4). Higher scores represent greater psychological distress. The fainter, thinner lines and points show patient-level data. The more opaque, thicker lines show the means for each randomized group.

Illness perception, as measured by the BIPQ (whereby a lower score reflects a less threatening view of illness), was statistically significantly reduced in the intervention group compared to the control group (*Table 2*) (*Figure 1*). At 6 months, the mean ± standard deviation (SD) BIPQ scores in the intervention and control group were 39.5 ± 14.8 (−9.6 ± 17.0 change from baseline) vs. 47.6 ± 13.7 (0.4 ± 12.8 change from baseline) [adjusted mean difference: −8.67 (95% CI −12.04, −5.30; *P* < 0.001)]. This improvement in illness perception was also evident at 12 months (36.9 ± 15.2 (−12.2 ± 16.7 change from baseline) vs. 47.0 ± 14.2, (−0.2 ± 13.6 change from baseline) [adjusted mean difference: −10.80 (95% CI −14.27, −7.33); *P* < 0.001].

**Table 2:** Illness perception and psychological patient reported outcome measures

	Control *n* = 126		Intervention *n* = 124		Intervention effect: estimate
Timepoint	Follow-up value	Change from baseline	Follow-up value	Change from baseline	(95% CI), *P*-value
**BIPQ**					
6 months Mean (SD)	47.6 (13.7)	0.4 (12.8)	39.5 (14.8)	−9.6 (17.0)	−8.67 (−12.04, −5.30), *P* < 0.001
12 months Mean (SD)	47.0 (14.2)	−0.2 (13.6)	36.9 (15.2)	−12.2 (16.7)	−10.80 (−14.27, −7.33), *P* < 0.001
**PHQ4 (anxiety score)**					
6 months Mean (SD)	0.7 (1.0)	0.1 (0.6)	0.2 (0.6)	−0.5 (1.1)	−0.49 (−0.66, −0.32), *P* < 0.001
12 months Mean (SD)	0.7 (1.1)	0.0 (0.6)	0.2 (0.6)	−0.6 (1.1)	−0.51 (−0.68, −0.34), *P* < 0.001
**PHQ4 (depression score)**					
6 months Mean (SD)	0.5 (1.0)	0.2 (0.6)	0.2 (0.5)	−0.3 (1.0)	−0.38 (−0.55, −0.21), *P* < 0.001
12 months Mean (SD)	0.5 (1.0)	0.2 (0.7)	0.1 (0.4)	−0,4 (1.0)	−0.45 (−0.63, −0.28), *P* < 0.001
**PHQ4 (total score)**					
6 months Mean (SD)	1.2 (1.9)	0.2 (1.0)	0.4 (1.0)	−0.9 (2.0)	−0.88 (−1.19, −0.57) *P* < 0.001
12 months Mean (SD)	1.2 (2.0)	0.2 (1.1)	0.3 (0.8)	−1.0 (1.9)	−0.97 (−1.29, −0.65) *P* < 0.001

In each case, a linear regression intervention effect estimate for the 6-month and 12-month values of the score is presented. This estimate is adjusted for the baseline value of the outcome, age, sex, diabetes, prior myocardial infarction, coronary artery disease, LV systolic function, and site (lead vs. other).

### Psychological health

Psychological health outcomes, assessed by the PHQ4, also favoured a stress-CMR guided strategy. Total PHQ-4 scores decreased to a greater extent in the intervention group compared with the control group at both 6 and 12 months, reflecting lower overall psychological distress. This treatment effect was evident for both the anxiety score and depression score, measured by PHQ4 (*Table 2*) (*Figure 1*).

At 6 months, the mean ± standard deviation (SD) PHQ4 total scores in the intervention and control group were 0.4 ± 1.0 (−0.9 ± 2.0 change from baseline) vs. 1.2 ± 1.9 (0.2 ± 1.0 change from baseline) [adjusted mean difference: −0.88 (95% CI −1.19, −0.57); *P* < 0.001] and these differences were also evident at 12 months (0.3 ± 0.8 (−1.0 ± 1.9 change from baseline) vs. 1.2 ± 2.0, (0.2 ± 1.1 change from baseline) [adjusted mean difference: −0.97 (95% CI −1.29, −0.65); *P* < 0.001].

### Heterogeneity of intervention effect by common demographic factors and endotype

Estimates for interactions between the effect of the CMR intervention and demographic factors such as age, sex, deprivation status, and endotypes are described in the Supplement (see Supplementary material online, *Tables S3–S12*).

There was evidence of interactions of deprivation status, reflected by the Scottish Index of Multi-deprivation (SIMD), on the effect of the CMR intervention on BIPQ scores at 6 and 12 months (*P* = 0.048 and *P* = 0.043, respectively; Supplementary material online, *Table S5*), and PHQ4 scores at 6 and 12 months (*P* = 0.015 and *P* = 0.007, respectively; Supplementary material online, *Table S10*) indicating less deprivation associates with a greater reductions in illness perception and psychological distress by this CMR strategy. Considering endotypes, the CMR intervention also reduced BIPQ and PHQ4 scores more at these timepoints in patients with a final CMR diagnosis of microvascular angina (see Supplementary material online, *Tables S6* and *S11*), but this was not the case for patients with non-cardiac chest pain (see Supplementary material online, *Tables S7* and *S12*).

There was no evidence of an interaction between age tertile and sex, and the effects of the intervention on BIPQ or PHQ4 were measured at 6 and 12 months.

## Discussion

In this randomized, multicentre trial of patients with chest pain, a strategy of non-invasive endotyping using stress perfusion CMR, linked to endotype-informed medical therapy, resulted in substantial and sustained improvements in illness perception and psychological health (central illustration). The intervention group demonstrated clinically meaningful gains in both domains: more favourable illness perceptions as measured by the Brief Illness Perception Questionnaire (BIPQ) and reduced symptoms of anxiety and depression as assessed by the Patient Health Questionnaire-4 (PHQ-4). These findings highlight that diagnostic clarification and endotype-specific management address not only ischaemic symptoms but also the substantial psychological burden experienced by this high symptom burden patient population.

The study design involved longitudinal assessments over time, including at 6 and 12 months, in order to assess the time-course of any effect of the intervention and specifically whether an effect might be sustained in the longer term. One of the most relevant findings for participant well-being was the magnitude and durability of improvement in illness perception, as measured by the BIPQ. Participants in the intervention group experienced marked reductions in total BIPQ score, reflecting more coherent and less threatening perceptions of their condition.

Illness perception is a central determinant of outcomes in chronic disease, influencing symptom burden, coping behaviours, treatment adherence, and psychological well-being.^6,18^ Maladaptive illness beliefs, including perceptions of poor understanding and lack of control, have been associated with greater symptom persistence, impaired quality of life, and worse mental health outcomes in patients with stable angina and related syndromes.^2,3,19^ In ANOCA, where coronary angiography often excludes obstructive disease without providing an alternative mechanistic explanation, diagnostic uncertainty may exacerbate these maladaptive perceptions.

Our findings support the concept that providing an accurate diagnosis through non-invasive functional endotyping can fundamentally reshape how patients conceptualize their illness. The durability of improvement to 12 months argues against a transient reassurance effect and instead suggests sustained cognitive reframing of illness beliefs. These findings are consistent with prior invasive strategy trials, including CorMicA and ILIAS-ANOCA, in which invasive functional endotyping was associated with improvements in patient-reported outcome measures, including illness perception.^20,21^

The observed improvements in psychological health, encompassing both anxiety and depression symptoms as assessed by the PHQ-4, complement these findings. Psychological distress is highly prevalent in patients with ANOCA and may both result from and contribute to symptom burden through complex biopsychosocial mechanisms.^2,22^ The bidirectional relationship between chest pain and psychological symptoms has been well documented, with anxiety potentially lowering pain thresholds and increasing symptom reporting, while persistent unexplained symptoms generate further anxiety and depression.^23,24^ By providing diagnostic clarity and effective symptom control, endotype-guided management may interrupt this cycle.

The parallel improvements observed in BIPQ and PHQ-4 outcomes suggest a close relationship between illness perception and psychological well-being in ANOCA. More favourable illness perception, characterized by improved understanding and greater perceived control, has been associated with lower levels of anxiety and depression in cardiovascular and other chronic disease populations.^6,18,19^ While causality cannot be definitively established, the consistency of effects across both domains supports a conceptual model in which diagnostic clarification positively influences patients’ understanding of their illness, with subsequent improvements in psychological health.

The CMR intervention had differential effects on illness perception and psychological distress according to deprivation status, with people living in areas with relatively less social deprivation appearing to gain greater benefits. We do not have information to provide a causal explanation for this observation. Multiple differences in income, employment, health, education, housing, access to primary and secondary care services, and exposure to crime may be relevant.

We also observed an interaction on the effect of the CMR-informed intervention by diagnosis, in that the improvements in illness perception and anxiety and depression by 6 and 12 months post-randomization occurred in patients with a final diagnosis of microvascular angina, rather than in individuals with a diagnosis of non-cardiac chest pain. One explanation for this finding is the beneficial effects of mechanism-informed angina therapy, including lifestyle measures and medicines known to benefit patients with microvascular angina, whereas this would not be the case for individuals with non-cardiac chest pain.

Our study has clinical implications, as adverse illness perceptions and psychological distress have been linked to worse cardiovascular prognosis and increased healthcare utilization in patients with chronic coronary syndromes.^2,4^ Our findings indicate that non-invasive CMR-guided endotyping provides a viable alternative pathway that addresses both physical and psychological dimensions of this syndrome.

Integration of functional endotyping into routine clinical pathways may reduce unnecessary repeat testing, emergency department visits, and hospitalizations driven by diagnostic uncertainty and inadequately controlled symptoms. Beyond healthcare utilization, the psychological benefits observed in our trial have intrinsic value for patient well-being and may contribute to improved cardiovascular outcomes through enhanced treatment adherence, healthier lifestyle behaviours, and favourable effects on biological pathways linking psychological distress to cardiovascular risk^25–27^

Considering limitations, while the PHQ-4 is a validated screening tool for anxiety and depression, it does not provide a detailed diagnostic assessment of psychiatric disorders. More comprehensive psychiatric assessment tools would provide additional insights into the mental health benefits of endotyping-guided care. Furthermore, longer-term follow-up beyond 12 months is needed to determine whether improvements in illness perception and psychological health are maintained, whether they translate into reduced cardiovascular events or healthcare utilization, and whether they predict long-term treatment adherence and quality of life. The relationship between psychological improvements and clinical outcomes warrants investigation in future studies with extended follow-up. In addition, objective diagnosis of coronary vasospasm is facilitated by invasive acetylcholine provocation testing, and this approach should be considered in patients with refractory symptoms. The protocol-directed prioritization of calcium channel blocker therapy for microvascular angina may have offset this diagnostic gap in patients with mixed microvascular/vasospastic angina. However, some patients with a diagnosis of non-cardiac chest pain may have had a missed diagnosis of isolated vasospastic angina. No adjustment has been made for multiple testing. Since the randomized diagnostic intervention was linked with medical therapy options and lifestyle measures, this stratified medicine approach limits the interpretation of mediating mechanisms and dose-response relationships.

In conclusion, in patients with angina and no obstructive coronary artery disease, non-invasive stress perfusion CMR used to guide endotype-specific management resulted in sustained improvements in illness perception and psychological health. These findings underscore the central importance of diagnostic clarity and demonstrate that functional endotyping can meaningfully improve how patients understand and psychologically experience their illness. Integrating non-invasive diagnostic endotyping into contemporary care pathways for ANOCA has the potential to address a major unmet psychological and perceptual burden in this population.

## Supplementary Material

qcag081_Supplementary_Data

## Data Availability

Data requests will be considered by the Sponsor and the University of Glasgow, who will take account of the scientific rationale, ethics, logistics, and resource implications. Data access requests should be initially submitted by email to the Chief Investigator (Colin Berry, corresponding author) and requesters can be expected to wait 4 weeks before the corresponding author informs them of the suitability of the request and if any additional information is required. The source data includes the deidentified numerical data used for the statistical analyses and deidentified imaging scans (MRI). Data access will be provided through the secure analytical platform of the Robertson Centre for Biostatistics. This secure platform enables access to deidentified data for analytical purposes, without the possibility of removing the data from the server. Requests for transfer of deidentified data (including source imaging scans) will be considered by the University of Glasgow, and if approved, a collaboration agreement would be expected, taking account of any cost implications. Cost recovery would be expected on a not-for-profit basis.

## Pre-registered clinical trial number

The pre-registered clinical trial number is Clinicaltrials.gov  **identifier**: NCT04805814 (https://clinicaltrials.gov/study/NCT04805814).
