# Supplementary material for: Endotype-informed therapy and effects on illness perception and psychological health in patients with chest pain and no obstructive coronary artery disease
Source: Eur Heart J Qual Care Clin Outcomes. 2026 Jun 4;12(6):998–1008. doi: 10.1093/ehjqcco/qcag081 (PMC13579331; doi:10.1093/ehjqcco/qcag081)
Supplement: Supplementary file 1 [file qcag081_supplementary_data.docx]

**Supplementary Material**

**Endotype-Informed Therapy and Effects on Illness Perception and Psychological Health in Patients with Chest Pain and No Obstructive Coronary Artery Disease**

Methods

# Stress/rest perfusion CMR imaging

CMR imaging was performed at 1.5 Tesla (Siemens MAGNETOM Avanto, Erlangen, Germany) using a standardized protocol. All participants were invited to abstain from caffeine-containing beverages or foodstuffs for 24-hours and vasoactive medications for 48-hours prior to the CMR examination.

The imaging protocol included localizers, cine imaging for cardiovascular dimensions and function including long axis left ventricular imaging e.g. 4- and 3-chamber acquisitions, aortic cine and flow sequences, short-axis planes through the left ventricle (basal, mid-ventricular and apical) for native myocardial longitudinal relaxation time (T1, ms) mapping and transverse relaxation time (T2, ms) mapping (mid-LV only) before intravenous administration of gadolinium contrast media, then followed by intravenous adenosine stress- and rest- myocardial perfusion imaging, short axis cine for left ventricular function, late gadolinium enhancement imaging and post- contrast myocardial T1 mapping.

Vasodilator stress was achieved by intravenous infusion of adenosine at a dose of 140μg/kg/min for 4 min (increased to 210μg/kg/min for a further 2 minutes if no symptoms or less than 10% heart rate increase). Splenic switch-off was retrospectively confirmed during image analysis to assess for adequate stress. At peak stress, a gadolinium-based contrast media (Gadovist®, Bayer Healthcare) was injected at 4 ml/s at a dose of 0.05 mmol/kg. Resting first-pass contrast (0.05 mmol/kg, Gadovist®) myocardial perfusion imaging was then performed at least ten minutes later. An intravenous top-up dose of contrast media (0.1 mmol/kg, Gadovist®) was given to optimize late gadolinium enhancement imaging.

# CMR analysis

The CMR imaging data were reviewed and reported by a Level 3 accredited imaging cardiologist at the Golden Jubilee University National Hospital, as per local standards of care. The CMR analyses were undertaken as per standard reporting guidelines ^1^ using commercially available software (CVI42, Circle Cardiovascular Imaging, Calgary, Canada). Left ventricular volumes and function were analyzed using manual planimetry. Late gadolinium enhancement was reported (17 segment model) with scores of 0 (no hyperenhancement), 1 (1–25% extent), 2 (26–50%), 3 (51–75%), or 4 (>75%). A positive result was taken as ≥2 adjacent segments (or 60^○^ arc-equivalent if the defect crosses segmental boundaries) with ≥50% transmural extent of ischemia, scar, or ischemia-scar combination) by protocol. Incidental findings identified during this review were referred for interpretation by a radiologist with further action according to standard care.

Automated quantitative myocardial blood flow mapping was performed using the method described by Kellman et al, including the Gadgetron framework ^2^. The method involves a dual sequence approach for myocardial perfusion acquisition and arterial input function acquisition simultaneously, allowing for quantification of myocardial blood flow (ml/min/g) for each pixel of left ventricular myocardium. The software allows for automated endocardial and epicardial contouring and segmentation using the American Heart Association 16- and 32- segment model. Automated endocardial and epicardial sub-segmentations are achieved by offsetting the epicardial border to 50%. The global myocardial blood flow is automatically calculated by the average of all the pixels and is measured at stress and rest. Automated contouring was reviewed and quality-checked by the reporting cardiologist. If errors were noted, automated contouring was removed and replaced by manual contours.

Global myocardial perfusion reserve (MPR) is the ratio of stress to rest myocardial blood flow. MPR can also be calculated specifically for the subendocardial layer (MPR_ENDO_) (calculated by stress MBF_ENDO_/ rest MBF_ENDO_). In keeping with prior studies, in the absence of a regional or visual perfusion defect, a threshold of global stress myocardial blood flow <2.25ml/min/g, MPR<2.2 or MPR_ENDO_<2.41 was deemed consistent with a diagnosis of coronary microvascular dysfunction^3,4^.

**Definitions of endotypes**

The diagnostic groups at the end of the standard care coronary angiogram were obstructive coronary artery disease, microvascular angina, vasospastic angina, and non-cardiac chest pain. This diagnosis was established by the attending interventional cardiologist based on the medical history, and results of noninvasive tests and the coronary angiogram. Following CMR imaging, the initial diagnosis post-angiography was re-evaluated by the imaging cardiologist for all participants based on the myocardial blood flow and incidental findings.

The myocardial blood flow results were used by the imaging cardiologist to define the true endotype documented in the research database according to diagnostic criteria^3,4^. In the intervention group, these findings were used to revise the final diagnosis and stratify patients into sub-groups (endotypes: microvascular angina, vasospastic angina, non-cardiac chest pain, obstructive coronary artery disease). In the control group, the myocardial blood flow results were not used to revise the final diagnosis.

Microvascular angina was defined according to the Coronary Vasomotion Disorders International Study Group (COVADIS) criteria^5^: symptoms of myocardial ischemia, unobstructed coronary arteries, and evidence of microvascular dysfunction. For the purposes of this clinical trial, microvascular dysfunction was defined by myocardial blood flow and MPR criteria based on CMR imaging.

During standard care invasive management, the interventional cardiologist may have performed adjunctive tests of coronary function. A diagnosis of microvascular spasm required provocation and reproduction of anginal symptoms, ischemic ECG shifts, but no epicardial spasm during acetylcholine testing^5^. Normal test results denoted no obstructive epicardial coronary artery disease (FFR>0.80) and no coronary vascular dysfunction (coronary flow reserve (CFR)>2.0, index of microvascular resistance (IMR)<25, and negative acetylcholine testing). A diagnosis of vasospastic angina required that three conditions occur during acetylcholine testing: (i) clinically significant epicardial vasoconstriction (≥90%) (ii) reproduction of the usual chest pain and (iii) ischemic changes on the electrocardiogram (ECG)^6^.

Tables

**Table 1.** Blinding effectiveness.

|  |  | All |  | Control | Intervention |
| --- | --- | --- | --- | --- | --- |
| N with pre-randomization diagnosis | | | | | |
| N |  | 250 |  | 126 | 124 |
| N randomised | | | | | |
| N |  | 250 |  | 126 | 124 |
| N with MRI diagnosis at MRI visit | | | | | |
| N |  | 249 |  | 126 | 123 |
| Clinician informed of randomized group allocation? | | | | | |
| N (N_MISSING_) |  | 245 (5) |  | 124 (2) | 121 (3) |
| N (%) Yes |  | 1 (0.4%) |  | 1 (0.8%) | 0 (0.0%) |
| Clinician aware of randomized group allocation (if not informed)? | | | | | |
| N (N_MISSING_) |  | 244 (0) |  | 123 (0) | 121 (0) |
| N (%) Yes |  | 0 (0.0%) |  | 0 (0.0%) | 0 (0.0%) |
| Participant informed of randomized group allocation? | | | | | |
| N (N_MISSING_) |  | 245 (5) |  | 124 (2) | 121 (3) |
| N (%) Yes |  | 1 (0.4%) |  | 1 (0.8%) | 0 (0.0%) |
| Participant aware of randomized group allocation (if not informed)? | | | | | |
| N (N_MISSING_) |  | 244 (0) |  | 123 (0) | 121 (0) |
| N (%) Yes |  | 0 (0.0%) |  | 0 (0.0%) | 0 (0.0%) |

**Table S2.** Differences between original diagnosis (based on clinical history and invasive coronary angiogram) and final diagnosis based on additional information following cardiovascular magnetic resonance imaging. The 'final working diagnosis’ (bold) was used to guide treatment decisions after randomization.

|  |  |  | Post-cardiovascular magnetic resonance imaging diagnosis | | | | |  |
| --- | --- | --- | --- | --- | --- | --- | --- | --- |
| Control N= 126 |  |  | Obstructive CAD | Microvascular angina | Vasospastic angina | Non-cardiac chest pain | Other diagnosis | **Final working diagnosis (control group)** |
|  | Original diagnosis | Obstructive CAD | 0 | 1 | 0 | 0 | 0 | **1 (0.8%)** |
|  |  | Microvascular angina | 0 | 1 | 0 | 0 | 0 | **1 (0.8%)** |
|  |  | Vasospastic angina | 0 | 0 | 0 | 0 | 0 | **0 (0.0%)** |
|  |  | Non-cardiac chest pain | 0 | 63 | 1 | 57 | 2 | **124 (98.4%)** |
|  |  | Other | 0 | 0 | 0 | 0 | 0 | **0 (0.0%)** |
|  |  |  | 0 (0.0%) | 65 (51.6%) | 1 (0.8%) | 57 (45.2%) | 2 (1.6%) |  |
| Intervention N= 124 |  |  | Obstructive CAD | Microvascular angina | Vasospastic angina | Non-cardiac chest pain | Other diagnosis |  |
|  | Original diagnosis | Obstructive CAD | 0 | 1 | 0 | 0 | 0 | 1 (0.8%) |
|  |  | Microvascular angina | 0 | 1 | 0 | 2 | 0 | 3 (2.4%) |
|  |  | Vasospastic angina | 0 | 0 | 0 | 0 | 0 | 0 (0.0%) |
|  |  | Non-cardiac chest pain | 0 | 60 | 0 | 58 | 2 | 120 (96.8%) |
|  |  | Other | 0 | 0 | 0 | 0 | 0 | 0 (0.0%) |
|  |  | **Final working diagnosis (intervention group)** | **0 (0.0%)** | **62 (50.0%)** | **0 (0.0%)** | **60 (48.4%)** | **2 (1.6%)** |  |
| CAD – coronary artery disease. | | | | | | | | |

| **Table S3.** Brief Illness Perception Questionnaire subgroup analysis - age tertile. | | |
| --- | --- | --- |
| The model for the BIPQ secondary outcome analysis is extended to include terms which estimate the interaction between treatment effect and subgroups defined by age tertiles. The interaction p-value assesses if there is an interaction effect. | | |
| Subgroup: age tertile | Change in BIPQ score: Median (Q1, Q3) | Intervention effect estimate (95% CI), p-value |
| Timepoint: 6 months | | |
| Tertile 1: 37 - 59 | -1.00 (-14.00, 3.00) | -12.26 (-17.88, -6.64), p<0.001 |
| Tertile 2: 60 - 68 | 0.00 (-9.75, 4.00) | -6.51 (-12.46, -0.57), p=0.032 |
| Tertile 3: 69 - 85 | -1.00 (-8.25, 4.00) | -6.75 (-12.86, -0.64), p=0.031 |
| Interaction p-value: | | p=0.294 |
| Timepoint: 12 months | | |
| Tertile 1: 37 - 59 | -3.50 (-16.25, 2.00) | -14.64 (-20.40, -8.87), p<0.001 |
| Tertile 2: 60 - 68 | -3.50 (-10.75, 3.75) | -9.58 (-15.67, -3.48), p=0.002 |
| Tertile 3: 69 - 85 | -2.50 (-10.00, 2.00) | -7.51 (-13.78, -1.24), p=0.019 |
| Interaction p-value: | | p=0.235 |

| **Table S4.** Brief Illness Perception Questionnaire subgroup analysis - sex. | | |
| --- | --- | --- |
| The model for the BIPQ secondary outcome analysis is extended to include terms which estimate the interaction between treatment effect and subgroups defined by sex. The interaction p-value assesses if there is an interaction effect. | | |
| Subgroup: sex | Change in BIPQ score: Median (Q1, Q3) | Intervention effect estimate (95% CI), p-value |
| Timepoint: 6 months | | |
| Male | -1.00 (-11.25, 4.00) | -10.14 (-14.91, -5.37), p<0.001 |
| Female | -1.00 (-9.00, 3.00) | -7.29 (-12.04, -2.55), p=0.003 |
| Interaction p-value: | | p=0.406 |
| Timepoint: 12 months | | |
| Male | -4.00 (-15.00, 3.00) | -13.06 (-17.94, -8.18), p<0.001 |
| Female | -3.00 (-11.00, 2.00) | -8.56 (-13.42, -3.71), p=0.001 |
| Interaction p-value: | | p=0.200 |

| **Table S5.** Brief Illness Perception Questionnaire subgroup analysis - SIMD group. | | |
| --- | --- | --- |
| The model for the BIPQ secondary outcome analysis is extended to include terms which estimate the interaction between treatment effect and subgroups defined by SIMD quintile. The interaction p-value assesses if there is an interaction effect. | | |
| Subgroup: SIMD group | Change in BIPQ score: Median (Q1, Q3) | Intervention effect estimate (95% CI), p-value |
| Timepoint: 6 months | | |
| Quintiles 1 and 2 | 0.00 (-10.00, 4.00) | -12.43 (-17.30, -7.56), p<0.001 |
| Quintiles 3, 4, and 5 | -1.50 (-10.00, 3.00) | -5.49 (-10.33, -0.65), p=0.026 |
| Interaction p-value: | | p=0.048 |
| Timepoint: 12 months | | |
| Quintiles 1 and 2 | -3.00 (-13.50, 4.00) | -15.10 (-20.04, -10.15), p<0.001 |
| Quintiles 3, 4, and 5 | -4.00 (-13.00, 1.00) | -7.89 (-12.81, -2.98), p=0.002 |
| Interaction p-value: | | p=0.043 |

| **Table S6.** Brief Illness Perception Questionnaire subgroup analysis - MRI diagnosis of microvascular angina. | | |
| --- | --- | --- |
| The model for the BIPQ secondary outcome analysis is extended to include terms which estimate the interaction between treatment effect and subgroups defined by an MRI diagnosis of MICROVASCULAR ANGINA. The interaction p-value assesses if there is an interaction effect. | | |
| Subgroup: MRI diagnosis of MICROVASCULAR ANGINA | Change in BIPQ score: Median (Q1, Q3) | Intervention effect estimate (95% CI), p-value |
| Timepoint: 6 months | | |
| Yes | -2.00 (-12.00, 5.00) | -14.14 (-18.82, -9.46), p<0.001 |
| No | -0.50 (-9.00, 2.75) | -3.18 (-7.92, 1.57), p=0.189 |
| Interaction p-value: | | p=0.001 |
| Timepoint: 12 months | | |
| Yes | -5.00 (-13.50, 3.00) | -16.78 (-21.57, -11.99), p<0.001 |
| No | -2.00 (-11.00, 2.00) | -4.74 (-9.60, 0.11), p=0.056 |
| Interaction p-value: | | p=0.001 |

| **Table S7.** Brief Illness Perception Questionnaire subgroup analysis - MRI diagnosis of non-cardiac chest pain. | | |
| --- | --- | --- |
| The model for the BIPQ secondary outcome analysis is extended to include terms which estimate the interaction between treatment effect and subgroups defined by an MRI diagnosis of non-cardiac chest pain. The interaction p-value assesses if there is an interaction effect. | | |
| Subgroup: MRI diagnosis of non-cardiac chest pain | Change in BIPQ score: Median (Q1, Q3) | Intervention effect estimate (95% CI), p-value |
| Timepoint: 6 months | | |
| Yes | 0.00 (-8.00, 3.00) | -1.65 (-6.44, 3.13), p=0.497 |
| No | -2.00 (-12.25, 4.25) | -15.19 (-19.71, -10.67), p<0.001 |
| Interaction p-value: | | p<0.001 |
| Timepoint: 12 months | | |
| Yes | -2.00 (-11.00, 2.00) | -3.20 (-8.09, 1.69), p=0.198 |
| No | -5.00 (-14.25, 2.25) | -17.80 (-22.42, -13.18), p<0.001 |
| Interaction p-value: | | p<0.001 |

| **Table S8.** Patient Health Questionnaire subgroup analysis - age tertile. | | |
| --- | --- | --- |
| The model for the PHQ-4 secondary outcome analysis is extended to include terms which estimate the interaction between treatment effect and subgroups defined by age tertiles. The interaction p-value assesses if there is an interaction effect. | | |
| Subgroup: age tertile | Change in PHQ-4 score: Median (Q1, Q3) | Intervention effect estimate (95% CI), p-value |
| Timepoint: 6 months | | |
| Tertile 1: 37 - 59 | 0.00 (0.00, 0.00) | -0.93 (-1.45, -0.41), p<0.001 |
| Tertile 2: 60 - 68 | 0.00 (0.00, 0.00) | -0.60 (-1.14, -0.05), p=0.032 |
| Tertile 3: 69 - 85 | 0.00 (0.00, 0.00) | -1.12 (-1.68, -0.56), p<0.001 |
| Interaction p-value: | | p=0.407 |
| Timepoint: 12 months | | |
| Tertile 1: 37 - 59 | 0.00 (-1.00, 0.00) | -0.90 (-1.42, -0.37), p=0.001 |
| Tertile 2: 60 - 68 | 0.00 (0.00, 0.00) | -0.69 (-1.24, -0.13), p=0.016 |
| Tertile 3: 69 - 85 | 0.00 (-1.00, 0.00) | -1.35 (-1.93, -0.78), p<0.001 |
| Interaction p-value: | | p=0.246 |

| **Table S9.** Patient Health Questionnaire subgroup analysis - sex. | | |
| --- | --- | --- |
| The model for the PHQ-4 secondary outcome analysis is extended to include terms which estimate the interaction between treatment effect and subgroups defined by sex. The interaction p-value assesses if there is an interaction effect. | | |
| Subgroup: sex | Change in PHQ-4 score: Median (Q1, Q3) | Intervention effect estimate (95% CI), p-value |
| Timepoint: 6 months | | |
| Male | 0.00 (0.00, 0.00) | -1.08 (-1.52, -0.63), p<0.001 |
| Female | 0.00 (0.00, 0.00) | -0.72 (-1.16, -0.28), p=0.001 |
| Interaction p-value: | | p=0.268 |
| Timepoint: 12 months | | |
| Male | 0.00 (-1.00, 0.00) | -1.18 (-1.63, -0.73), p<0.001 |
| Female | 0.00 (0.00, 0.00) | -0.80 (-1.25, -0.35), p=0.001 |
| Interaction p-value: | | p=0.246 |

| **Table S10.** Patient Health Questionnaire subgroup analysis - SIMD group. | | |
| --- | --- | --- |
| The model for the PHQ-4 secondary outcome analysis is extended to include terms which estimate the interaction between treatment effect and subgroups defined by SIMD quintile. The interaction p-value assesses if there is an interaction effect. | | |
| Subgroup: SIMD group | Change in PHQ-4 score: Median (Q1, Q3) | Intervention effect estimate (95% CI), p-value |
| Timepoint: 6 months | | |
| Quintiles 1 and 2 | 0.00 (0.00, 0.00) | -1.27 (-1.71, -0.83), p<0.001 |
| Quintiles 3, 4, and 5 | 0.00 (0.00, 0.00) | -0.49 (-0.93, -0.05), p=0.028 |
| Interaction p-value: | | p=0.015 |
| Timepoint: 12 months | | |
| Quintiles 1 and 2 | 0.00 (0.00, 0.00) | -1.41 (-1.86, -0.95), p<0.001 |
| Quintiles 3, 4, and 5 | 0.00 (0.00, 0.00) | -0.52 (-0.97, -0.07), p=0.025 |
| Interaction p-value: | | p=0.007 |

| **Table S11.** Patient Health Questionnaire subgroup analysis - MRI diagnosis of microvascular angina. | | |
| --- | --- | --- |
| The model for the PHQ-4 secondary outcome analysis is extended to include terms which estimate the interaction between treatment effect and subgroups defined by an MRI diagnosis of MICROVASCULAR ANGINA. The interaction p-value assesses if there is an interaction effect. | | |
| Subgroup: MRI diagnosis of MICROVASCULAR ANGINA | Change in PHQ-4 score: Median (Q1, Q3) | Intervention effect estimate (95% CI), p-value |
| Timepoint: 6 months | | |
| Yes | 0.00 (-1.00, 0.00) | -1.63 (-2.06, -1.21), p<0.001 |
| No | 0.00 (0.00, 0.00) | -0.14 (-0.57, 0.29), p=0.511 |
| Interaction p-value: | | p<0.001 |
| Timepoint: 12 months | | |
| Yes | 0.00 (-1.00, 0.00) | -1.82 (-2.25, -1.39), p<0.001 |
| No | 0.00 (0.00, 0.00) | -0.15 (-0.59, 0.28), p=0.488 |
| Interaction p-value: | | p<0.001 |

| **Table S12.** Patient Health Questionnaire subgroup analysis - MRI diagnosis of non-cardiac chest pain. | | |
| --- | --- | --- |
| The model for the PHQ-4 secondary outcome analysis is extended to include terms which estimate the interaction between treatment effect and subgroups defined by an MRI diagnosis of non-cardiac chest pain. The interaction p-value assesses if there is an interaction effect. | | |
| Subgroup: MRI diagnosis of non-cardiac chest pain | Change in PHQ-4 score: Median (Q1, Q3) | Intervention effect estimate (95% CI), p-value |
| Timepoint: 6 months | | |
| Yes | 0.00 (0.00, 0.00) | -0.10 (-0.54, 0.34), p=0.647 |
| No | 0.00 (-1.00, 0.00) | -1.61 (-2.03, -1.20), p<0.001 |
| Interaction p-value: | | p<0.001 |
| Timepoint: 12 months | | |
| Yes | 0.00 (0.00, 0.00) | -0.12 (-0.56, 0.33), p=0.603 |
| No | 0.00 (-1.00, 0.00) | -1.79 (-2.22, -1.37), p<0.001 |
| Interaction p-value: | | p<0.001 |

**Figures**

**Figure 1.** Clinician Questionnaire

**Figure 2.** Illness Perception and Psychological Health Outcomes at Baseline, 6 months, and 12 months.

The estimated treatment effect in units is stated with 95% confidence intervals at 6 and 12 months (intervention group and control group). **(A)** Illness Perception: Brief Illness Perception Questionnaire (BIPQ). Higher scores represent more negative perceptions of illness. **(B)**Psychological Health: Patient Health Questionnaire-4 (PHQ-4). Higher scores represent greater psychological distress

**References**

1.	Kramer CM, Barkhausen J, Bucciarelli-Ducci C, Flamm SD, Kim RJ, Nagel E. Standardized cardiovascular magnetic resonance imaging (CMR) protocols: 2020 update. *J Cardiovasc Magn Reson* 2020;**22**:17.

2.	Kellman P, Hansen MS, Nielles-Vallespin S, Nickander J, Themudo R, Ugander M, et al. Myocardial perfusion cardiovascular magnetic resonance: optimized dual sequence and reconstruction for quantification. *Journal of Cardiovascular Magnetic Resonance* 2017;**19**:43.

3.	Kotecha T, Martinez-Naharro A, Boldrini M, Knight D, Hawkins P, Kalra S, et al. Automated Pixel-Wise Quantitative Myocardial Perfusion Mapping by CMR to Detect Obstructive Coronary Artery Disease and Coronary Microvascular Dysfunction. *JACC Cardiovasc Imaging* 2019;**12**:1958–1969.

4.	Rahman H, Scannell CM, Demir OM, Ryan M, McConkey H, Ellis H, et al. High-Resolution Cardiac Magnetic Resonance Imaging Techniques for the Identification of Coronary Microvascular Dysfunction. *JACC Cardiovasc Imaging* 2021;**14**:978–986.

5.	Ong P, Camici PG, Beltrame JF, Crea F, Shimokawa H, Sechtem U, et al. International standardization of diagnostic criteria for microvascular angina. *Int J Cardiol* 2018;**250**:16–20.

6.	Beltrame JF, Crea F, Kaski JC, Ogawa H, Ong P, Sechtem U, et al. International standardization of diagnostic criteria for vasospastic angina. *Eur Heart J* 2017;**38**:2565–2568.
